# The impact of mandatory waiting periods on abortion-related outcomes: a synthesis of legal and health evidence

**DOI:** 10.1186/s12889-022-13620-z

**Published:** 2022-06-21

**Authors:** Fiona de Londras, Amanda Cleeve, Maria I. Rodriguez, Alana Farrell, Magdalena Furgalska, Antonella Lavelanet

**Affiliations:** 1 Birmingham Law School, University of Birmingham (UK), B15 2TT, Birmingham, UK; 2 Women’s and Children’s Health, Karolinska Institute, Stockholm, Sweden; 3 Department of Sexual and Reproductive Health and Research, UNDP-UNFPA-UNICEF-WHO-World Bank Special Programme of Research, Development and Research Training in Human Reproduction (HRP), World Health Organization, Geneva, Switzerland; 4 Department of Obstetrics and Gynecology, Oregon Health and Science University, Portland, OR USA; 5 York Law School, University of York (UK), York, UK

**Keywords:** Abortion, Mandatory waiting periods, Cooling off periods, Reflection periods, Reproductive rights, Sexual and reproductive health

## Abstract

This review follows an established methodology for integrating human rights to address knowledge gaps related to the health and non-health outcomes of mandatory waiting periods (MWPs) for access to abortion. MWP is a requirement imposed by law, policy, or practice, to wait a specified amount of time between requesting and receiving abortion care. Recognizing that MWPs “demean[] women as competent decision-makers”, the World Health Organization recommends against MWPs. International human rights bodies have similarly encouraged states to repeal and not to introduce MWPs, which they recognize as operating as barriers to accessing sexual and reproductive healthcare. This review of 34 studies published between 2010 and 2021, together with international human rights law, establishes the health and non-health harms of MWPs for people seeking abortion, including delayed abortion, opportunity costs, and disproportionate impact. Impacts on abortion providers include increased workloads and system costs.

**Supplementary Information:**

The online version contains supplementary material available at 10.1186/s12889-022-13620-z.

## Background

A mandatory waiting period (MWP) is a requirement imposed by law, policy, or practice, to wait a specified amount of time between requesting and receiving abortion care [1]. This is additional to the delays or waits that are generally built into the provision of non-emergency health care, including abortion, within health systems. While MWPs are not common, they are mandated by law and policy in several national and sub-national jurisdictions [2]. These MWPs vary widely across different settings [3]. In some cases, they can be satisfied in one visit, with the ‘clock’ beginning to run when telephone or other remote contact is made with a provider. In other settings mandatory waiting periods operate as ‘two visit’ requirements, with an in-person visit being required before the time begins to run. Some jurisdictions vary the application of MWP by gestational age. Sometimes referred to as ‘waiting periods’, ‘reflection periods’ or ‘cooling-off periods’ the World Health Organization (WHO) has recognized that MWPs “demean[] women as competent decision-makers” ([4], pg. 96). Reflecting this, the WHO recommends against MWPs [1]. International human rights bodies have similarly encouraged states to repeal and not to introduce MWPs, which they recognize as operating as barriers to accessing sexual and reproductive healthcare [5].

The aim of this review is to address knowledge gaps related to the health and non-health outcomes of MWPs. The review followed a methodology for integrating human rights in guideline development that has been described elsewhere [6]. This methodological approach is well-suited to interventions that are complex and can have multiple components interacting synergistically or dissynergistically, may be non-linear in their effects, and are often context dependent [7]. Such complex interventions often interact with one another so that outcomes related to one individual or community may be dependent on others, and may be impacted positively or negatively by the people, institutions and resources and how they are arranged within the larger system in which they are implemented [7]. As such, this review is not a classic systematic review per se but rather aims to synthesize evidence from existing studies (i.e. data extracted from included studies) and international human rights law (i.e. standards articulated in and by international human rights law sources and bodies) according to a methodology designed for this purpose [6]. This review was conducted as part of the evidence base for the WHO’s Abortion Care Guideline (2022) [1]. It is one of seven such reviews undertaken by the same research team and pursuing a common methodology.

Throughout this review we use the terms women, pregnant women, women and girls, and pregnant people interchangeably to refer to all those who are or can become pregnant, regardless of their gender identity.

## Methods

### Identification of studies and data extraction

This review examined the impact of the intervention of MWPs on two populations: (i) people seeking abortion, and (ii) healthcare providers. Legal, policy, and human rights experts co-developed the study outcomes and search strategy. Our outcomes of interest included both health and non-health outcomes that, based on a preliminary assessment of the literature [8], could be linked to the effects of MWPs. Our a priori outcomes included delayed abortion, opportunity costs, self-managed abortion, workload implications, system costs, perceived imposition on personal ethics or conscience, perceived impact on relationship with patient, referral to another provider, unlawful abortion, continuation of pregnancy, and stigmatization.

Our search strategy contained a combination of MeSH and key words. We searched the databases PubMed, HeinOnline, JStor, and the search engine Google Scholar. As the second edition of the WHO’s Safe Abortion Guidance included data up until 2010, we limited our search to papers published in English after 31 December 2010 and up to 2 December 2019. An updated search of the same databases was undertaken in July 2021. We did not restrict our search by study design. We included (comparative and non-comparative) quantitative studies, qualitative and mixed-methods studies, reports, PhD theses, and economic or legal analyses that undertook original data collection or analysis, but excluded masters theses and abstracts.

The full review team was made up of 6 members (MF, AF, FdL, AC, MR and AL). AL and FdL developed the PICO. Two reviewers (MF and AF) conducted an initial screening of the literature. Titles and abstracts were first screened for eligibility using the Covidence® tool; full texts were then reviewed. A third reviewer (FdL) confirmed that these manuscripts met inclusion criteria. Two reviewers (FdL and AC) extracted data. Any discrepancies were reviewed and discussed with two additional reviewers (AL and MR). The review team resolved discrepancies through consensus.

In accordance with our previously-published methodology for the effective integration of human rights as evidence in systematic reviews for guideline development [6], we identified and analyzed international human rights law as it related to reproductive rights in order to identify relevant human rights standards. Once data had been extracted from the included studies, we integrated them with the identified human rights standards. This allowed us to develop a full understanding of the implications of MWPs abortion law and policy including (a) which human rights standards are engaged by MWPs, (b) whether the studies suggest that MWPs have positive or negative effects on the enjoyment of rights, and (c) where no data is identified from the manuscripts against outcomes of interest, whether human rights law provides evidence that can further elucidate the impacts and effects of MWPs. This is summarized in Tables 2 and 3 below.

### Analysis

We organized data from the included studies by reference to our study outcomes and presented this in evidence tables. These tables presented the association of each study on the outcome together with an overall conclusion from the data relevant to the outcome of interest. We then applied human rights standards to these outcomes to develop an understanding of the effects of criminalization that combines the evidence from human rights law (i.e. the applicable standards) and the included studies. In other words, we assessed whether the evidence from the included studies indicated that MWPs had effects that were incompatible with established requirements of international human rights law [6]. To summarize the effect of the intervention, across all study designs, we used and applied a visual representation of effect direction. The direction of the evidence was illustrated by a symbol which indicated whether, in relation to that particular outcome, the evidence extracted from a study suggested an increase (▲), decrease (⊽), or no change in the outcome (○). The symbol did not indicate the magnitude of the effect [6].

## Results

**Fig. 1:**
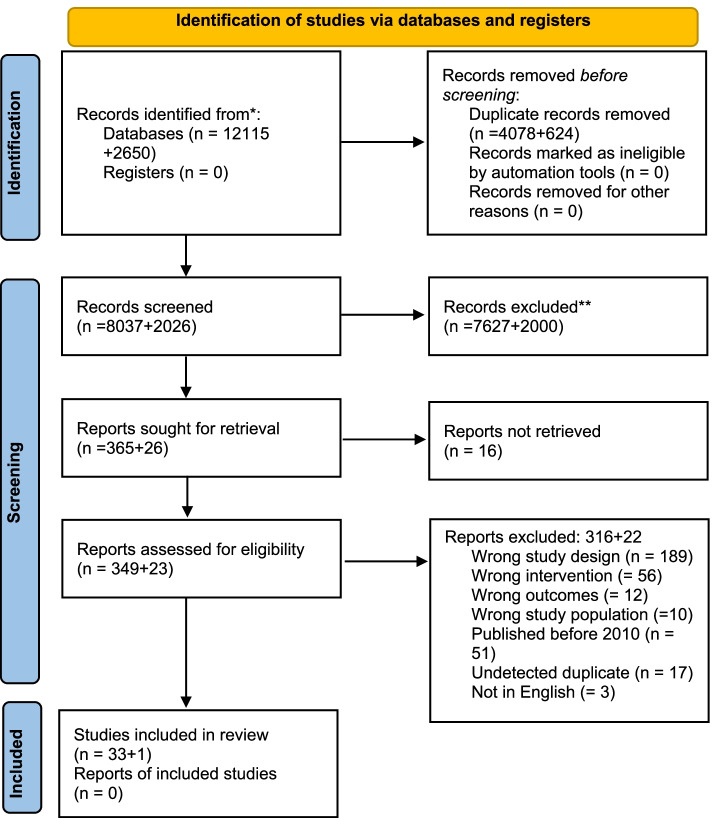
Prisma Flow diagram. *Consider, if feasible to do so, reporting the number of records identified from each database or register searched (rather than the total number across all databases/registers). **If automation tools were used, indicate how many records were excluded by a human and how many were excluded by automation tools

The search generated 10,063 citations after duplicates were removed. We screened the titles and abstracts and conducted a full text screening of 391 manuscripts. We excluded those manuscripts that did not have a clear connection with the intervention and our pre-defined outcomes, resulting in 34 manuscripts being included in the final analysis (Fig. 1. Prisma flow diagram).

All manuscripts described data from the United States of America [9–42]. The characteristics of included manuscripts are presented in Table 1. The included studies contained information relevant for the outcomes: delayed abortion [14, 15, 19, 30, 31, 40–42], continuation of pregnancy [9, 10, 22–25, 27, 29, 35, 37, 41, 42], opportunity costs [14, 15, 9, 11–13, 16–18, 20, 21, 25, 26, 28, 32–35, 40, 41], disproportionate impact [9, 21, 37, 40–42], workload implications [30], and system costs [9, 26, 22, 36, 35, 37, 38, 41, 39]. No evidence was identified linking the intervention to the outcomes unlawful abortion, self-managed abortion, disqualification from lawful abortion, referral to another provider, stigmatization of providers, and impact on the provider-patient relationship.

**Table 1:** Characteristics of included studies

Author/year	Country	Methods	Participants/data
Coles 2010 [9]	United States of America	Retrospective cohort study (*n* = 8245) using data from the Pregnancy Risk Assessment Monitoring System.	Self-reported data reported by women giving birth across 30 states over a 6-year period.
Colman 2010 [10]	Texas, United States of America	Time series design (*n* = 16,029).	State level data on abortions occurring over a 7-year period.
Cooney 2017 [11]	United States of America	Cross sectional study (*n* = 286).	Genetic counsellors with prenatal experiences with a mean of 8.7 years’ experience.
Dennis 2014 [12]	Oklahoma, Arizona and Kansas, United States of America	Qualitative individual interviews (*n* = 30).	Women with experiences of abortion in three states.
Ely 2019 [13]	Tennessee, United States of America	Cross sectional study (*n* = 422).	Women seeking abortion services in the state from one abortion provider.
Ehrenreich 2019a^1^ [14]	Utah, United States of America	Qualitative individual interviews (*n* = 18).	Women aged 18–40 years using telemedicine to attend state-mandated information visits.
Ehrenreich 2019b^1^ [15]	Utah, United States of America	Qualitative individual interviews (*n* = 20).	Women accessing abortion services, some of who opted for an information visit by telemedicine.
Fuentes 2019 [16]	United States of America	Cross sectional study (*n* = 11,024) using the Abortion Patient Survey, 2014.	Women obtaining an abortion at 87 healthcare facilities.
Jerman 2017^1^ [17]	Michigan and. New Mexico, United States of America	Qualitative individual interviews (*n* = 29).	Women aged 18–44 seeking abortion services at 6 facilities who had travelled across state lines or more than 100 miles within a state.
Jones 2013 [18]	United States of America	Cross sectional study (*n* = 8338) using data from the Abortion Patient Survey, 2008.	Women obtaining abortions at 95 facilities in 34 different states.
Jones 2016 [19]	United States of America	Cross sectional study (*n* = 7414) using the Abortion Patient Survey, 2014.	Women obtaining an abortion at 87 “non-hospital” healthcare facilities.
Jones 2017 [20]	v	Cross sectional study (*n* = 8380) using the Abortion Patient Survey, 2014.	Women obtaining an abortion at 87 “non-hospital” healthcare facilities
Karasek 2016 [21]	Arizona, United States of America	Cross sectional study (*n* = 379).	Women aged 18–45 obtaining an abortion at one healthcare facility.
Medoff 2010a [22]	United States of America	Time series design (*n* = not reported).	Multiple data sources: Data on non-marital birth-rates from Centers for Disease Control; economic data from the US Census of Population, 2003.
Medoff 2010b [23]	United States of America	Time series design (*n* = not reported).	Multiple data sources: abortion data from Guttmacher Institute; socio-economic data from the US Bureau of the Census and the Statistical Abstract of the United States.
Medoff 2012 [24]	United States of America	Time series design (*n* = not reported).	Multiple data sources: abortion data from the Guttmacher Institute; socio-economic data from State Reports of the U. S Census Bureau.
Medoff 2014a [25]	United States of America	Time series design (*n* = not reported).	Multiple data sources: abortion data from Centers for Disease Control and Guttmacher Institute; socio-economic data from Statistical Abstract of the Unites States.
Medoff 2014b [26]	United States of America	Time series design (*n* = not reported).	Multiple data sources: data on pregnancy intentions from Centers for Disease Control; data on births from the US Vital Statistics Report.
Medoff 2014c [27]	United States of America	Time series design (*n* = not reported).	Multiple data sources: data on unintended pregnancy from a previous publication; abortion data from the Guttmacher Institute.
Medoff 2015 [28]	United States of America	Time series design (*n* = not reported).	Multiple data sources: abortion data from the Guttmacher Institute; data on number of healthcare providers from the US Bureau of the Census, Statistical Abstract of the United States.
Medoff 2016 [29]	United States of America	Time series design (*n* = not reported).	Abortion data from Guttmacher Institute; data on unintended births from a previous publication.
Mercier 2015^1^ [30]	North Carolina, United States of America	Qualitative individual interviews (*n* = 31).	Abortion providers (physicians, nurses, physician assistant, counselor and clinic administrators) working under the Women’s Right to Know Act (WRKA) with previous experience of working in a less restrictive environment.
Morse 2018^2^ [31]	North Carolina, United States of America	Cross sectional study (*n* = 48).	Women seeking an abortion at one healthcare facility over a 16-week period, some before and some after the waiting period was changed from 24 to 72 hours.
Myers 2021 [42]	United States of America	Randomized control trial, different in differences and event study (Poisson model).	Data from various sources including CDC abortion surveillance data, Guttmacher Institute statistics, NCHS data on state-level birth counts, state level estimates from SEER.
Roberts 2016 [32]	Utah, United States of America	Prospective cohort study (*n* = 500).	Women presenting at an abortion information visit at one healthcare facility.
Roberts 2017 [33]	Utah, United States of America	Prospective cohort study (*n* = 500).	Women presenting at an abortion information visit at one healthcare facility.
Ruhr 2016 [34]	Missouri, United States of America	Mixed methods study (*n* = 139/52 completed follow up survey).	Women 18 years and older seeking an abortion for an unintended pregnancy.
Sanders 2016 [35]	Utah, United States of America	Cross sectional study (*n* = 3618 from database/307 completed questionnaire).	Abortion data from 11 clinics before and after the waiting period was changed from 24 h to 72 h. Women seeking abortion at a healthcare facility after the 72 h-law came into effect.
Sen 2012 [36]	United States of America	Time series design (*n* = 5100).	Data from Centers for Disease Control and Prevention/National Center for Health Statistics Multiple Cause of Death public-use data files, 1983–2002, on deaths among children 0–4 years old.
Tosh 2015 [37]	United States of America	Cross sectional study (*n* = not reported).	State level population data from 50 states.
Wallace 2017 [38]	United States of America	Cross sectional study (*n* = 3,948,761).	Data from multiple sources Data on live births in 2011 were obtained from The National Center for Health Statistics.
White 2016^1^ [40]	Alabama, United States of America	Qualitative individual interviews (*n* = 25).	Women aged 19 years and above seeking abortion at two clinics after travelling more than 30 miles one way.
White 2017 [41]	Alabama, United States of America	Cross sectional study (*n* = 2730).	Billing data from two clinics for all abortions over a 12-month period.
Williams 2018 [39]	Arizona, United States of America	Time series design (*n* = 43,692).	Data from multiple sources including: Demographic and Health Survey data, before and after legislation of abortion restrictions came into effect

### Impact of the intervention on abortion seekers

A summary of the impacts of the intervention on abortion seekers and the application to human rights are presented in Table 2. Evidence identified per study and outcome are presented in Supplementary Table 1.

**Table 2:** Overall conclusions from Table A, PICO 1 + Summary B-table + Conclusion from C-table

Outcome	Overall conclusion of evidence (A)	Application of HR standards (B)	Conclusion evidence + HR (C)
Delayed abortion	Overall, evidence from 8 studies suggest that MWPs contribute to abortion delays by increasing the time from counselling to the abortion appointment, and by contributing to logistical difficulties in obtaining care. This effect is magnified when two visits are required.	MWPs engage states’ obligation to respect, protect and fulfil the rights to life and health (by taking steps to reduce maternal mortality and morbidity including addressing unsafe abortion, and protecting people seeking abortion).	MWPs can result in delayed access to abortion care. Where such delays increase risks of maternal mortality or morbidity, they have negative implications for rights.
Continuation of pregnancy	Overall, evidence from 6 studies suggest that MWPs may contribute to the continuation of pregnancy, especially among adolescents, Black, and Hispanic women, women who have to travel far for an abortion, and poor women. The effect is greater where two visits are required rather than one. Evidence from 7 studies suggest that MWPs do not contribute to any changes to abortion rates, unintended pregnancy or birth rates in general, but MWPs may decrease births among unmarried women.	MWPs engage states’ obligation to respect, protect and fulfil the rights to life and health (by ensuring abortion regulation is evidence-based and proportionate), the right to equality and non-discrimination, and the right to decide on the number and spacing of one’s children.	Where MWPs are associated with undesired continuation of pregnancy they may interfere disproportionately with the rights of abortion seekers. This may disproportionately be the case for adolescents, Black, and Hispanic women, women who have to travel far for an abortion and poor women.
Opportunity costs	Overall, evidence from 18 studies suggest that MWPs contribute to opportunity costs including financial and emotional impacts such as: logistical burdens, emotional stress, financial costs, increased prices for abortions, increased travel time, and out of state travel. Online or phone-based counselling may mitigate some opportunity costs related with two-visits. The negative impacts of MWPs are exacerbated for women who need to travel far for an abortion. Evidence from 2 studies suggest that MWPs are not associated with incidence of postpartum depression and for most women, MWPs do not impact women’s certainty in the abortion decision.	MWPs engage states’ obligation to respect, protect and fulfil the rights to life and health (by ensuring abortion regulation is evidence-based and proportionate, and that where it is lawful abortion is safe and accessible), and the right to equality and non-discrimination.	MPWs are associate with opportunity costs. These costs (including travel costs, unnecessary multiple visits.) make abortion less accessible in practice, and are exacerbated for women who need to travel for abortion.
Unlawful abortion	No evidence identified.	MWPs engage states’ obligation to respect, protect and fulfil the rights to life and health (by taking steps to reduce maternal mortality and morbidity including addressing unsafe abortion, and protecting people seeking abortion).	The operation of MWPs may lead persons to avail of abortions outside of the formal medical system, including unlawful abortions. Such abortions may be unsafe. States must take steps to reduce maternal mortality and morbidity, including addressing unsafe abortion. Disqualification from lawful abortion as a result of the application of a MWP (often in conjunction with gestational limits) can result in criminal liability when a pregnant person seeks abortion outside the formal system, including availing of unlawful self-managed abortion. Criminalisation of abortion may constitute a human rights violation.
SMA	No evidence identified.	MWPs engage states’ obligation to respect, protect and fulfil the rights to life and health (by taking steps to reduce maternal mortality and morbidity including addressing unsafe abortion, and protecting people seeking abortion).	The operation of MWPs may lead persons to avail of abortions outside of the formal medical system, including self-managed abortions. Such abortions may be unsafe. States must take steps to reduce maternal mortality and morbidity, including addressing unsafe abortion. Disqualification from lawful abortion as a result of the application of a MWP (often in conjunction with gestational limits) can result in criminal liability where a pregnant person seeks abortion outside the formal system including availing of unlawful self-managed abortion. Criminalisation of abortion may constitute a human rights violation.
Disqualification from lawful abortion	No evidence identified.	MWPs engage states’ obligation to respect, protect and fulfil the rights to life and health (by taking steps to reduce maternal mortality and morbidity including addressing unsafe abortion, and protecting people seeking abortion). They may also result in the violation of the state’s obligation to ensure abortion is available where the life and health of the pregnant person is at risk, or where carrying a pregnancy to term would cause her substantial pain or suffering, including where the pregnancy is the result of rape or incest or where the pregnancy is not viable.	MWPs may result in women exceeding gestational limits, which may result in disqualification from lawful abortion including in cases of sexual violence or therapeutic abortion, with implications for the rights to health, life, security of person, and privacy. Disqualification from lawful abortion as a result of the application of a MWP (often in conjunction with gestational limits) can result in criminal liability where a person avails of abortion without satisfaction of the MWP. Criminalisation of abortion may result in a violation of the right to equality and non-discrimination, right to security of person, or right to be free from torture, and cruel, inhuman and degrading treatment.
Disproportionate impact	Overall, evidence from 6 studies suggest that MWPs have a disproportionate negative impact on women who need to travel farther for an abortion, women of colour, and women with fewer resources.	MWPs engage states’ obligation to respect, protect and fulfil the right to equality and non-discrimination.	MWPs have a disproportionate impact on women of colour, women with fewer resources, and women who need to travel for an abortion, with negative implications for the right to equality and non-discrimination in the provision of sexual and reproductive healthcare.
Referral to another provider	No evidence identified.	MWPs engage states’ obligation to respect, protect and fulfil the rights to life and health (by taking steps to reduce maternal mortality and morbidity including addressing unsafe abortion, and protecting people seeking abortion).	MWPs may operate to delay referral and thus delay access to abortion care.

Evidence from six studies suggests that MWPs contribute to abortion delays [15, 19, 40, 31, 41, 42], including in waiting times for appointments [15, 19] and for women who need to travel to access abortion [40], with delays being greater where MWPs are longer (72 hours compared to 24 hours [31], for example) or where they require two visits [41, 42]. These delays are in excess of the MWP itself. In some cases MWPs cause delays that limit available abortion management options [14]. The delays associated with MWPs can be increased where the MWP is combined with mandated scripted counselling, provision of which requires the reorganization of physician time [30]. The right to the maximum attainable standard of physical and mental health (‘right to health’) requires that reproductive care be available, accessible and of good quality [43]. Such delays, which are attributable to a law and policy intervention and not, for example, to resource scarcity, raise questions of compatibility of MWPs with the right to health. This is exacerbated by the expectation in human rights law that abortion regulation would be evidence-based and proportionate [44], and the requirement not to regulate abortion in a way that violates women’s and girls’ right to life, jeopardizes their lives, subjects them to physical or mental pain or suffering, discriminates against them, or arbitrarily interferes with their privacy [45]. Given this, MWPs are prima facie disproportionate as a matter of human rights law.

Evidence on the effect of MWPs on continuation of pregnancy is mixed. Seven studies suggest that MWPs do not contribute to any change to abortion rates [22], unintended pregnancy rates [24, 27] or birth rates [29] in general, one of which suggests MWPs are associated with decreased non-marital birth rates [22]. However, evidence from six studies suggests that MWPs may contribute to continuation of pregnancy through increased birth rates [42], decreased abortion rates [25], or failure to return for the second visit [35]. Where MWPs are associated with continuation of pregnancy studies showed differential impacts depending on age [9, 37], race or ethnicity [25, 37], resources [41], and distance travelled [41] to access abortion. In studies where no effect on birth rates was detected, the MWPs did not require an in-person visit [10], or was part of a multi-part TRAP law which imposed multiple restrictions [23]. The evidence from five studies suggests that MWPs impose disproportionate burdens across sub-populations of people seeking abortion. Evidence from three studies suggests that Black and Hispanic women, especially minors [37] and younger women [42], are particularly impacted by MWPs [9, 37, 42], while other studies suggest that there are disproportionate burdens for women who have fewer resources [21, 41, 42] and have to travel to access abortion care [21, 40–42].

While the evidence of the effect of MWPs on continuation of pregnancy is mixed, it is clear that where such effects exist they impact disproportionately on identifiable sub-populations. This aligns with the broader evidence from this review on the disproportionate impact of MWPs. This is inconsistent with the right to equality and non-discrimination, as well as the right to health. The United Nations Working Group on the issue of discrimination against women in law and in practice has noted that “[b] arriers to accessing lawful abortion that are not based on medical needs … are discriminatory” [46]. MWPs fall into this classification.

Evidence from twenty studies suggests that MWPs contribute to opportunity costs [14, 15, 9, 11–13, 16–18, 20, 21, 25, 26, 28, 32–35, 40, 41] for people who seek abortion. Studies found that abortion seekers and providers perceive MWPs as restricting care [14, 11] or making abortion seem unattainable [21], contributing to emotional [14, 12, 32, 34] and logistical burdens [14] including abortion travel [17], additional visits [15], delays [15, 40, 20, 33], increased travel time [15, 13], distance [16, 18] and costs [15, 13, 28, 32, 34, 35, 40], and unwanted disclosure of pregnancy [35]. Evidence from two studies suggest that MWPs are not associated with incidence of postpartum depression [26] and for most women, MWPs do not impact women’s certainty in the abortion decision [33]. Such opportunity costs reduce in practice the availability of abortion and thus have negative implications for the right to health.

### Impact of the intervention on healthcare providers

A summary of the impacts of the intervention on healthcare providers and the application to human rights are presented in Table 3. Evidence identified per study and outcome are presented in Supplementary Table 2.

**Table 3:** Overall conclusions from Table A, PICO 2 + Summary B-table + Conclusion from C-table

Outcome	Overall conclusion of evidence (A)	Application of HR standards (B)	Conclusion evidence + HR (C)
Workload implications	Overall, evidence from 1 study suggests that MWPs, including when the first visit can be done by phone, contribute to workload implications by increasing staffing costs and logistical difficulties.	MWPs engage states’ obligation to respect, protect and fulfil the rights to life and health (by ensuring abortion regulation is evidence-based and proportionate, and by protecting healthcare professionals providing abortion care).	Workload implications arising from MWPs place significant burdens on healthcare professionals providing abortion care and may result in reduced or hindered access to abortion with negative implications for both their rights and the rights of persons seeking to access abortion.
System costs	Overall, evidence from 4 studies suggests that MWPs contribute to system costs by: increasing child homicides and unwanted births among minors (Black minors in particular) and by decreasing the proportion of abortions performed < 14 weeks and by decreasing medication abortions. Evidence from 2 studies suggest that when women cannot return for an abortion procedure due to MWPs, the impact on system costs is unclear. Evidence from 2 studies suggest that MWPs do not contribute to system costs relating to preterm birth, low birth weight or postpartum depression, and evidence from 1 study indicates that MWPs reduce system costs by lowering non-marital births.	MWPs engage states’ obligation to respect, protect and fulfil the rights to life and health (by ensuring abortion regulation is evidence-based and proportionate), and the right to equality and non-discrimination.	MWPs are associated with system costs. In the absence of clinical justification for such MWPs, these costs may constitute a disproportionate interference with the rights of abortion seekers. This may disproportionately be the case for adolescents and Black minors.
Stigmatization	No evidence identified.	MWPs engage states’ obligation to respect, protect and fulfil the rights to life and health (by protecting healthcare professionals providing abortion care).	N/A
Impact on provider-patient relationship	No evidence identified	N/A	N/A

Evidence from one study [30] suggests that MWPs contribute to increased workload, even where the first ‘visit’ or trigger for the waiting period can be done remotely, which may lead to additional staffing costs and logistical challenges. Importantly, this study considered a MWP that was combined with a requirement for mandated scripted counselling provided by a prescribed health professional. Identified workload implications should be understood in this light.

Evidence from four studies suggests that MWPs contribute to system costs. One study found that MWPs were associated with increased odds of reporting an unwanted birth among minors [9], while other studies identified an association with an increase in child homicides [36], racial disparities in teen birth rates [37], and (combined with other regulatory policies) a decrease in the proportion of medication abortions [39]. Evidence from further studies suggest that MWPs are not associated with any change in the incidence of postpartum depression [26], or with preterm birth [38]. One study found that MWPs were associated with a decrease in nonmarital birth rates [22]. In system cost terms, the studies suggest that fewer women return for an abortion after a 72-hour MWP [35] leading to increased continued pregnancy rates with system cost impacts, and that two-visit MWP requirements are associated with adolescents and women with fewer resources returning for the abortion.

## Discussion

As with most non-emergency health care provision, delays are built into the provision of abortion meaning that the imposition of additional MWPs is both harmful and unnecessary [47]. Policy-makers and legislators who support MWPs sometimes argue that they are designed to ensure and support certainty for women who seek abortion, and to minimize post-abortion regret. However, as a general matter, women who decide to end their pregnancies reach that decision quickly [48] and experience a high level of decisional certainty [49]. There is no significant increase in decisional certainty where an MWP is imposed [50], and more recent research reinforces the finding that MWPs delay abortion and impose opportunity costs on women [51], which in turn have disproportionate impacts on poor women and those who live further away from clinics [52]. Post-abortion regret is very rare. Instead, in the United States (where all the reviewed studies were set) post-abortion relief is the most commonly felt emotion among women five years after abortion [53], while emotional difficulty relating to abortion is rooted in social disapproval, romantic relationship loss, and ‘head versus heart’ conflict [54]. MWPs do not address and cannot aid in resolving these experiences. Indeed, they may exacerbate them by reinforcing perceptions of social disapproval. Where women are unsure or seek to revisit their decision, providers are well-equipped to support this through the provision of additional time [55]; legal or policy mandates requiring such a ‘reflection period’ are not necessary to ensure that women can reach a decision in the time that is right for them.

Human rights bodies have made it clear that states should repeal laws and policies that impede access to sexual and reproductive health care, including MWPs. They have noted the effects of MWPs on rural women [5] and identified MWPs as interventions that create barriers to access to sexual and reproductive health care [56]. The evidence from this review bears out that characterization of MWPs, which impose barriers to accessing care without clinical function or benefit. MWPs are also not justifiable as modes of managing resource scarcity. Indeed, their implications for health professionals’ workloads suggests they may have the opposite effect.

In addition, evidence identified in this review suggests that women who seek abortion broadly experience and perceive MWPs as burdensome, emotionally damaging, and negative in their effects. The UN Human Rights Committee has made clear that “[m] easures introduced to regulate abortion may not violate women’s and girls’ right to life, jeopardize their lives, subject them to physical or mental pain or suffering, discriminate against them, or arbitrarily interfere with their privacy” ([45], para. 8). This review suggests that MWPs fall foul of this requirement.

### Limitations

This review has limitations. The most obvious limitation is that all the studies reviewed took place in the United States. While some studies were set in the United States generally [9, 11, 16, 18–20, 22–29, 36, 37, 42, 38], others were conducted across one or more states, those being Alabama [40, 41], Arizona [12, 21, 39], Kansas [12] Michigan [17], Missouri [34], New Mexico [17], North Carolina [30, 31], Oklahoma [12], Tennessee [13], Texas [10], and Utah [14, 15, 32, 33, 35]. While the dynamics of abortion law and policy that apply in the United States are particular, the effects of MWPs as a law and policy intervention are not limited to this particular setting. Indeed, at national level most MWPs are contained in European countries’ laws [3]. Thus, research on MWPs and their impact on access to abortion in more settings would be welcome. In addition, MWPs are highly variable and the nature and severity of their effects may differ depending on, for example, how they are triggered (by an in person visit, by telephone consultation, or by completion of prescribed formalities, for example) or gestational age [3]. Research taking these variables into account would further enrich the evidence base. Furthermore, in several included studies MWPs were introduced as part of, or operated in the context of, a multi-part and complex law and policy intervention, including so-called TRAP laws [57]. Thus, while these studies considered the impact of MWPs this was in their broader regulatory context and, in some cases, as part of a broader consideration of the effects of a TRAP law per se. Although the methodology adopted in this review acknowledges the interactions of multiple interventions and seeks to understand the studied intervention in its context [6, 7], studies that specifically consider the impacts of MWPs in settings with such omnibus packages of restrictive law and policy interventions would likely be illuminating.

As a general matter, the realization of human rights applicable to abortion-related interventions is not a research area that readily lends itself to randomized controlled trials or comparative observational studies; rather, studies are often conducted without comparisons. While this may be considered a limitation from a standard methodological perspective for systematic reviews, it does not limit the ability to identify human rights law implications of law and policy interventions. Thus, while standard tools for assessing risk of bias or quality, including GRADE [58], or the use of plausibility as an inclusion criteria, were unsuitable, given the objective of fully integrating human rights implications into our understanding of the effects of provider restrictions as a regulatory intervention, it was appropriate to engage with a wide variety of sources. Similarly, given the methodological approach adopted [6] we did not use plausibility as an inclusion criteria.

## Conclusion

The evidence from the reviewed studies and international human rights law points clearly towards the inappropriateness of MWPs in abortion law and policy. As noted by the CESCR Committee, “[s]tates should repeal and refrain from introducing measures that create barriers to [sexual and reproductive health] goods and services” [56]. These include MWPs.

## Supplementary Information

**Additional file 1: Supplementary Table 1.** Evidence table: Impact on the intervention on abortion seekers. **Supplementary Table 2.** Evidence Table: The impact of the intervention on health professionals.

## Data Availability

All data generated or analyzed during this study are included in the published article and its supplementary information files.
