# Supplementary material for: The impact of mandatory waiting periods on abortion-related outcomes: a synthesis of legal and health evidence
Source: BMC Public Health. 2022 Jun 21;22:1232. doi: 10.1186/s12889-022-13620-z (PMC9210763; doi:10.1186/s12889-022-13620-z)
Supplement: Supplementary file 1 — Additional file 1: Supplementary Table 1. Evidence table: Impact on the intervention on abortion seekers. Supplementary Table 2. Evidence Table: The impact of the intervention on health professionals. [file 12889_2022_13620_MOESM1_ESM.docx]

Tables to be included as supplementary material:

Suppl. Table 1: Full B-table PICO 1

Suppl. Table 2: Full B-table PICO 2

*Supplementary Table 1. Evidence table: Impact on the intervention on abortion seekers*

| **OUTCOME: DELAYED ABORTION** | | | |  |
| --- | --- | --- | --- | --- |
| **Studies** | **Direction of the evidence** | **What does this mean?** | **Overall conclusion** |  |
| Ehrenreich 2019a^1^ | ▲ | Abortion seekers perceive that MWPs restrict access to care. For some women MWPs cause delays that limit the available abortion management options. | Overall, evidence from 8 studies suggest that MWPs contribute to abortion delays by increasing the time from counselling to the abortion appointment, and by contributing to logistical difficulties in obtaining care. This effect is magnified when two visits are required. |  |
| Ehrenreich 2019b^1^ | ▲ | MWPs add to the waiting time for an appointment and cause delays in accessing care |  |  |
| Jones 2016 | ▲ | - MWPs are associated with an increased time to AN abortion appointment of 1.5-2 days: without a two-visit requirement, mean days = 8.2 - with a two-visit requirement, mean days = 8.9 - no MWP, mean days = 6.7.   MWPs are associated with increased odds of making an abortion appointment >14 days following the initial visit compared with no MWPs:   - without a two-visit requirement- OR 1.45; 95% CI 1.07-1.98 - with two-visit requirement- OR 1.88; 95% CI 1.39-2.54. |  |  |
| Mercier 2015^1^ | ▲ | When MWPs are combined with mandated scripted counselling by a health professional, logistical difficulties in providing the care may further increase abortion delays. |  |  |
| Morse 2018^2^ | ▲ | A 72-hour MWP compared with a 24-hour MWP may increase time from first clinic visit to the abortion procedure. |  |  |
| Myers 2021 | ▲ | MWPs that require in-person visits reduce the proportion of abortions <9 weeks (8%) and increase proportion of second trimester abortions (one visit: 3.2%; two visits: 19%). |  |  |
| White 2016^1^ | ▲ | MWPs contribute to abortion delays among women who need to travel far for an abortion. |  |  |
| White 2017 | ▲ | Where MWPs with a two-visit requirement are implemented, women with fewer resources and women who need to travel farther (50-100 miles) for an abortion (OR 1.25; CI 95% 1.01-1.56) are more likely to have longer intervals between the two visits than women who travel less than 25 miles. |  |  |
| **OUTCOME: CONTINUATION OF PREGNANCY** | | | |  |
| Coles 2010 | ▲ | Among minors with live births, MWPs are associated with increased risk of reporting an unwanted birth (RR 2.51; 1.11-5.57). | Overall, evidence from 12 studies suggests that MWPs may contribute to continuation of pregnancy, especially among adolescents, Black, and Hispanic women. The effect is greater where the MWP requires two visits, especially for women of colour, women who need to travel far for an abortion, younger women, and women living in an area of socio-economic deprivation.  Evidence from 7 studies suggest that MWPs do not contribute to any changes to abortion rates, unintended pregnancy or birth rates in general, but MWPs may decrease births among unmarried women. |  |
| Colman 2010 | ○ | MWPs that do not require an in-person visit, but a self-attestation to having viewed mandatory online materials, are not associated with changes in abortion rates <16 weeks’ gestation after the policy change, or when compared with states without MWPs (mean 14.71/1000, SE 0.6, 4.11% change). |  |  |
| Medoff 2010a | ∇ | MWPs are associated with a 17% decrease in nonmarital birth rates among women aged 15-44 years. |  |  |
| Medoff 2010b | ○ | Targeted Regulation of Abortion Provider (TRAP) laws (state laws that impose licensing fees, physical/personnel regulations and other regulations on abortion providers’ governing procedures and protocols) that include MWPs, are not associated with significant changes in abortion rates. |  |  |
| Medoff 2012 | ○ | MWPs with a two-visit requirement are not associated with significant changes in unintended pregnancy rates. |  |  |
| Medoff 2014a | ○ | MWP with a two-visit requirement are associated with a significant decrease in abortion rates among white women, and no change among Latina or Black women. Regression coefficient:  White abortion rate: β = −0.035 (2.08), p <.05,  Black abortion rate: β = 1.1068 (0.5), p > 0.10,  Hispanic abortion rate: β = 0.3751 (0.77), p>0.10. |  |  |
| Medoff 2014c | ○ | MWPs are not associated with significant changes in unintended pregnancy rates. |  |  |
| Medoff 2016 | ○ | MWPs are not associated with changes in unintended birth rates. |  |  |
| Myers 2021 | ▲ | MWPs requiring two-visits increase birth rates by 1.5% and one-visit MWP by 0.5%. Two-visit MWPs are estimated to reduce abortion rates by state of occurrence by 10.2% and by state of residence by 8.8%. |  |  |
| Sanders 2016 | ▲ | Fewer women return for the abortion procedures after implementation of a 72-hour MWP (77% after implementation vs. 80% before p<0.5) and may instead continue their pregnancies. |  |  |
| Tosh 2015* | ▲ | Abortion regulations, including MWPs, are negatively associated with teen abortion rates (β = -.437, p=0.013) and positively associated with Black teen birth rates (β =.525, P=.008), but do not significantly affect white teen birth rates (β =. 219, p =.230 or Hispanic teen birth rates (β =.367, p=.068). |  |  |
| White 2017 | ▲ | Where MWPs with a two-visit requirement are implemented, women with fewer resources (OR 1.47; 95% CI 1.20-1.80) and those travelling 50-100 miles one way (OR 1.25; 95% CI 1.01-1.56) are more likely to have longer durations between the first and second visit. Adolescents (OR 2.97; 95% CI 1.40-6.27) and women with fewer resources (OR 1.51; 95% CI 1.19-1.92) are more likely not to return for an abortion, and may instead continue their pregnancies. |  |  |
| **OUTCOME: OPPORTUNITY COSTS** | | | |  |
| Ehrenreich 2019a^1^ | ▲ | Abortion seekers perceive that MWPs restrict access to care and contribute to emotional and logistical burdens. For some women MWPs cause delays that limit the options available for abortion management. | Overall, evidence from 20 studies suggest that MWPs contribute to opportunity costs including financial and emotional issues such as: logistical burdens, emotional stress, financial costs, increased prices for an abortion, increased travel time, and out of state travel. Online or phone-based counselling may mitigate some opportunity costs related with two-visits. The negative impacts of MWPs are exacerbated for women who need to travel far for an abortion.  Evidence from 2 studies suggest that MWPs are not associated with incidence of postpartum depression and for most women, MWPs do not impact women´s certainty in the abortion decision. |  |
| Ehrenreich 2019b^1^ | ▲ | MWPs are associated with opportunity costs; including an additional clinic visit, delays, increased travel time and financial costs, some of which may be mitigated through the use of telemedicine. |  |  |
| Coles 2010 | ▲ | Among minors with live births, MWPs are associated with reporting an unwanted birth (RR 2.51; 1.11-5.57). |  |  |
| Cooney 2017 | ▲ | Genetic counsellors perceive that MWPs restrict women’s access to abortion and places additional stress on abortion seekers. |  |  |
| Dennis 2014^1^ | ▲ | Women report that MWPs increase the emotional difficulty of abortion decisions and can have a negative impact. |  |  |
| Ely 2019 | ▲ | Where MWPs are implemented, geographical distance (to an abortion clinic) (B = –0.003, p= 0.151; 95% CI= –0.007 to 0.001) or rural residence (OR=1.013 95% CI=0.565 to 1.81) does not impact the likelihood of returning for the abortion. The travel, however, may be associated with opportunity costs including travel time and financial costs. |  |  |
| Fuentes 2019 | ▲ | Abortion seekers residing in settings with MWPs compared with no MWPs, may have higher odds of travelling further for an abortion (OR 1.7; 95% CI 1.0-2.8, p value 0.043). |  |  |
| Jerman 2017^1^ | ▲ | In an effort to avoid MWPs, abortion seekers may travel out of state as the MWPs are perceived as a barrier to accessing care. |  |  |
| Jones 2013 | ▲ | Women living in settings with MWPs compared with settings with no MWPs have higher odds of travelling further for abortion (OR 2.6; 95% CI 1.7-3.9). |  |  |
| Jones 2017 | ▲ | MWPs with a two-visit requirement compared to no MWPs, decrease the likelihood of obtaining an abortion <6 weeks (OR 0.51; 95% CI 0.39-1.66). MWPs do not increase the likelihood of a second trimester abortion. |  |  |
| Karasek 2016 | ▲ | Among women with economic hardships, MWPs are associated with increased odds of the perception that an abortion would not be attainable (OR 1.88, 95% CI 1.18-2.99). |  |  |
| Medoff 2014a | ▲ | MWPs with a two-visit requirement are associated with a significant decrease in abortion rates among white women, and no change among Latina or Black women.  Regression coefficient:  White abortion rate: β= −0.035 (2.08), p<.05  Black abortion rate: β = 1.1068 (0.5), p>0.10  Hispanic abortion rate: β= 0.3751 (0.77), p>0.10 |  |  |
| Medoff 2014b | ○ | MWPs are not associated with changes in the incidence of postpartum depression. |  |  |
| Medoff 2015 | ▲ | MWPs and a two-visit requirement are associated with an increase in the inflation-adjusted price for an abortion by 107 US dollars (19%) and a decrease in the number of abortions performed (between 13-15%). |  |  |
| Roberts 2016 | ▲ | A MWP with a two-visit requirement increases financial costs for women, with the first visit representing 11% of the abortion costs. For some women, the MWP and two-visit requirement, causes frustration, and emotional stress and anxiety. |  |  |
| Roberts 2017 | ▲ | A majority of women (455/500) report that the MWP does not impact certainty of their decision but results in a minimum of a 72-hour delay. |  |  |
| Ruhr 2016 | ▲ | A 72-hour MWP, compared with 24-hour MWP, is associated with a significantly larger number of days waiting for an abortion after signing the consent form (mean 7.6 vs. mean 6.1 days). 55/132 of the abortion seekers did not support the 72-hour MWP and perceive it as having a negative impact on them. Some women perceived the MWP as being an emotional, mental, and physical burden for them and reported increased financial costs due lost wages and travel costs. |  |  |
| Sanders 2016 | ▲ | Out of 307 women, 62% report that the 72-hour MWP impacted them negatively, including lost wages or needing to take extra time off work (47%), excess childcare costs (18%) increased transportation costs (30%), lost wages by family or friends (27%), and unwanted disclosure of pregnancy (33%). |  |  |
| White 2016^1^ | ▲ | For women who needed to travel far for an abortion, the MWPs with a two-visit requirement are especially burdensome as they are linked to increased travel costs, time off work, and abortion delays. |  |  |
| White 2017 | ▲ | Where MWPs with a two-visit requirement are implemented, women with fewer resources and women who need to travel farther for an abortion (OR 1.25; CI 95% 1.01-1.56) are more likely to have longer intervals between the two visits. |  |  |
| **OUTCOME: DISPROPORTIONATE IMPACT** | | | |  |
| Coles 2010 | ▲ | Hispanic and Black minors may be disproportionately impacted by MWPs. MWPs are associated with increased risk of mistimed birth among Hispanic minors (RR 2.86; p<0.1), and increased risk of unwanted birth among Black minors (RR 3.9; p<0.1). | Overall, evidence from 5 studies suggest that MWPs have a disproportionate negative impact on women who need to travel farther for an abortion, women of colour, and women with fewer resources. |  |
| Karasek 2016 | ▲ | Women who experienced more economic difficulties as a result of the abortion procedure (aOR 1.88; 95% CI 1.18-2.99) and those who experienced delays in obtaining an abortion (aOR 4.39 95% CI 1.11-17.42) are more likely to report that a MWP with a two-visit requirement would prevent them from accessing abortion care. Women who experience more economic trade-offs (aOR 1.50; 95% CI 1.11-1.93) and women who travel >1 hour (aOR 2.6; 95% CI 1.26-5.52) are also more likely to report that a MWP would further delay access to care. |  |  |
| Myers 2021 | ▲ | Where MWPs have an effect on birth rates, this effect disproportionately impacts people of colour, especially when requiring two visits, (2.7% increase for non-Hispanic black women versus 1.1% increase for white non-Hispanic women). The effect of MWPs disproportionately impact young women, women who have to travel far for an abortion and those who are poor; two-visit MWPs are estimated to increase births by 2.8% ages 15-19, 3.5% for ages 20-24, and 3.7% for ages 25-29, versus by 1.2% for ages 30-34; birth rates are estimated to increase among young women by 3.2% in counties with the lowest unemployment rates but by 4.7% in counties with the highest unemployment rates; birth rates for women <30 years are estimated to increase with increasing distance from a provider (by 2.3% for those with shortest distance and 7.8% for those with the longest distance). |  |  |
| Tosh 2015* | ▲ | Abortion restrictions, including MWPs, are negatively associated with teen abortion rates (β= -.437, p=0.013) and positively associated with Black teen birth rates (β=.525, P=.008), but do not significantly affect white teen birth rates (β=. 219, p =.230) or Hispanic teen birth rates (β=.367, p=.068). |  |  |
| White 2016^1^ | ▲ | For women who need to travel far for an abortion, an MWP with a two-visit requirement is especially burdensome, as it is linked to increased travel costs, time off work, and abortion delays. |  |  |
| White 2017 | ▲ | Where MWPs with a two-visit requirement are implemented, women with fewer resources (OR 1.47; 95% CI 1.20-1.80) and those travelling 50-100 miles one way (OR 1.25; 95% CI 1.01-1.56) are more likely to have longer durations between the first and second visits. Adolescents (OR 2.97; 95% CI 1.40-6.27) and women with fewer resources (OR 1.51; 95% CI 1.19-1.92) are more likely to not return for an abortion, which may lead to continuation of pregnancy. |  |  |

▲ = the intervention leads to an increase in the outcome; ○ = the intervention leads to no change in the outcome; ∇ = the intervention leads to a decrease in the outcome. Symbol does not indicate magnitude or certainty of effect

^1^ Qualitative study design: tests of statistical significance not applicable

^2^ Study was not powered to look at this outcome.

* Used an alpha of .10 to determine significance (p < 0.10)

*Supplementary Table 2. Evidence Table:* The impact of the intervention on health professionals

| **OUTCOME: WORKLOAD IMPLICATIONS** | | | |
| --- | --- | --- | --- |
| **Studies** | **Direction of the evidence** | **What does this mean?** | **Overall conclusion** |
| Mercier 2015^1^ | ▲ | Implementing MWPs, in combination with mandated scripted counselling by a health professional, is burdensome for health providers and organizations, as they increase staffing costs and require extensive changes to appointment schedules when two visits need to be organised. Even when consent and counselling can be provided over the phone, this requires staffing and bureaucratic changes. | Overall, evidence from 1 study suggest that MWPs, including when the first visit can be done over the phone, contribute to workload implications through increasing staffing costs and logistical difficulties. |
| **OUTCOME: SYSTEM COST** | | | |
| Coles 2010 | ▲ | MWPs are associated with increased odds of reporting an unwanted birth among minors with live births (RR 2.51; 95% CI 1.11-5.57). | Overall, evidence from 9 studies suggest that MWPs contribute to system costs by: increasing child homicides and unwanted births among minors, Black minors in particular; and by decreasing the proportion of abortions performed <14 weeks and by decreasing medication abortions.  Evidence from 2 studies suggest that when women cannot return for an abortion procedure due to MWPs, the impact on system costs is unclear.  Evidence from 2 studies suggests that MWPs do not contribute to system costs due to preterm birth, low birth weight or postpartum depression, and evidence from 1 study indicate that MWPs reduce system costs by lowering non-marital births. |
| Medoff 2014b | ○ | MWPs are not associated with changes in the incidence of postpartum depression. |  |
| Medoff 2010a | ∇ | MWPs are associated with a 17% decrease in non-marital birth rates among women 15-44 years. |  |
| Sen 2012 | ▲ | MWPs are associated with an increase in child homicides compared with settings without MWPs (OR 1.13; 95% CI 1.03-1.25). |  |
| Sanders 2016 | ○ | Fewer women return for abortion procedures after implementation of a 72-hour MWP (77% after implementation vs. 80% before p<0.5) and may instead continue their pregnancies. |  |
| Tosh 2015* | ▲ | Abortion regulations, including MWPs, are negatively associated with teen abortion rates (β= -.437, p=0.013) and positively associated with Black teen birth rates (β=.525, P=.008), but do not significantly affect white teen birth rates (β=. 219, p =.230 or Hispanic teen birth rates (β=.367, p=.068). |  |
| Wallace 2017 | ○ | MWPs are not associated with preterm birth (OR 1.06; 95% CI 0.96-1.18) or low birth weight (OR 1.01; 95% CI 0.93-1.09). |  |
| White 2017 | ○ | Where MWPs with a two-visit requiring are implemented, adolescents (OR 2.97; 95% CI 1.40-6.27) and women with fewer resources (OR 1.51; 95% CI 1.19-1.92) are more likely to not return for an abortion and may instead continue their pregnancies. |  |
| Williams 2018 | ▲ | When MWPs are implemented in combination with other regulatory policies, the proportion of medical abortions decreases by 17.8% (95% CI 16.9-18.8) and the proportion of abortions performed <14 weeks decreases by 3.3% (95% CI 2.8-3.8). |  |

▲ = the intervention leads to an increase in the outcome; ○ = the intervention leads to no change in the outcome; ∇ = the intervention leads to a decrease in the outcome. Symbol does not indicate magnitude or certainty of effect

^1^ Qualitative study design: tests of statistical significance not applicable

* Used an alpha of .10 to determine significance (p < 0.10)
